# Engaging Dementia Caregivers in Under-Resourced Areas to Examine Disparities in Post-Acute Care Quality and Access

**DOI:** 10.1093/geroni/igaa057.2774

**Published:** 2020-12-16

**Authors:** Andrea Gilmore-Bykovskyi, Laura Block

**Affiliations:** 1 University of Wisconsin-Madison, Madison, Wisconsin, United States; 2 University of Wisconsin-Madison School of Nursing, Madison, Wisconsin, United States

## Abstract

Dementia disproportionately impacts racial/ethnic minorities and individuals from under-resourced environments, yet these groups are under-represented in research. For caregivers, managing dementia often involves navigating frequent post-acute care (PAC) transitions. Despite evidence of segregation-associated disparities in PAC access and quality, the perspectives of caregivers from under-resourced areas regarding these disparities and how they are experienced, are poorly understood. We engaged a coalitional, community-informed approach to engaging caregivers in highly under-resourced areas to elicit experiences surrounding PAC through semi-structured interviews (N=25; 65% African American; 25% White; 88% female). Data were analyzed using thematic analysis. Caregivers spontaneously connected issues in PAC quality to racial/ethnic disparities and discrimination citing differences in geographic availability, financial barriers, eligibility, access to information, and transportation. To mitigate these challenges caregivers remained highly involved in the care recipient’s PAC, describing the need to continue to “advocate” and “supervise.” Collectively, these perspectives can help inform future, targeted policy interventions.

